# Exploring quantitative group-wise differentiation of Alzheimer’s disease and behavioural variant frontotemporal dementia using tract-specific microstructural white matter and functional connectivity measures at multiple time points

**DOI:** 10.1007/s00330-019-06061-7

**Published:** 2019-03-11

**Authors:** R. Meijboom, R. M. E. Steketee, L. S. Ham, D. Mantini, E. E. Bron, A. van der Lugt, J. C. van Swieten, M. Smits

**Affiliations:** 1 Department of Radiology and Nuclear Medicine, Erasmus MC – University Medical Centre Rotterdam, Rotterdam, The Netherlands; 2 Centre for Clinical Brain Sciences, University of Edinburgh, Edinburgh, United Kingdom; 3 Research Center for Motor Control and Neuroplasticity, KU Leuven, Leuven, Belgium; 4 Functional Neuroimaging Laboratory, IRCCS San Camillo Hospital Foundation, Lido, Italy; 5 Biomedical Imaging Group Rotterdam - Departments of Medical Informatics and Radiology, Erasmus MC - University Medical Centre Rotterdam, Rotterdam, The Netherlands; 6 Department of Neurology, Erasmus MC – University Medical Centre Rotterdam, Rotterdam, The Netherlands

**Keywords:** Alzheimer disease, Frontotemporal dementia, Functional MRI, Diffusion tensor imaging, Longitudinal studies

## Abstract

**Objectives:**

This study explored group-wise quantitative measures of tract-specific white matter (WM) microstructure and functional default mode network (DMN) connectivity to establish an initial indication of their clinical applicability for early-stage and follow-up differential diagnosis of Alzheimer’s disease (AD) and behavioural variant frontotemporal dementia (bvFTD).

**Methods:**

Eleven AD and 12 bvFTD early-stage patients and 18 controls underwent diffusion tensor imaging and resting state functional magnetic resonance imaging at 3 T. All AD and 6 bvFTD patients underwent the same protocol at 1-year follow-up. Functional connectivity measures of DMN and WM tract-specific diffusivity measures were determined for all groups. Exploratory analyses were performed to compare all measures between the three groups at baseline and between patients at follow-up. Additionally, the difference between baseline and follow-up diffusivity measures in AD and bvFTD patients was compared.

**Results:**

Functional connectivity of the DMN was not different between groups at baseline and at follow-up. Diffusion abnormalities were observed widely in bvFTD and regionally in the hippocampal cingulum in AD. The extent of the differences between bvFTD and AD was diminished at follow-up, yet abnormalities were still more pronounced in bvFTD. The rate of change was similar in bvFTD and AD.

**Conclusions:**

This study provides a tentative indication that quantitative tract-specific microstructural WM abnormalities, but not quantitative functional connectivity of the DMN, may aid early-stage and follow-up differential diagnosis of bvFTD and AD. Specifically, pronounced microstructural changes in anterior WM tracts may characterise bvFTD, whereas microstructural abnormalities of the hippocampal cingulum may characterise AD.

**Key Points:**

*• The clinical applicability of quantitative brain imaging measures for early-stage and follow-up differential diagnosis of dementia subtypes was explored using a group-wise approach.*

*• Quantitative tract-specific microstructural white matter abnormalities, but not quantitative functional connectivity of the default mode network, may aid early-stage and follow-up differential diagnosis of behavioural variant frontotemporal dementia and Alzheimer’s disease.*

*• Pronounced microstructural white matter (WM) changes in anterior WM tracts characterise behavioural variant frontotemporal dementia, whereas microstructural WM abnormalities of the hippocampal cingulum in the absence of other WM changes characterise Alzheimer’s disease.*

**Electronic supplementary material:**

The online version of this article (10.1007/s00330-019-06061-7) contains supplementary material, which is available to authorized users.

## Introduction

Presenile dementia is a dementia with an onset before the age of 65 years. The two most common underlying disorders are Alzheimer’s disease (AD) and behavioural variant frontotemporal dementia (bvFTD) [1]. AD is characterised by an episodic memory disturbance for recently learned as well as for learning new material, together with at least one other cognitive disturbance [2]. In contrast, bvFTD is mainly characterised by behavioural problems such as disinhibition, apathy and loss of empathy [3]. In later stages of AD and bvFTD, predominance of cognitive impairment in AD and social/executive impairment in bvFTD [4, 5] aids differential diagnosis. However, differential diagnosis can be difficult in early stages of AD and bvFTD, as symptoms may still be mild and unspecific. BvFTD patients may present with memory deficits [6, 7] and AD patients with changes in social behaviour or executive functioning [5, 7, 8]. Magnetic resonance imaging (MRI) supports diagnosis, but in early disease stages, conventional (structural) MRI may still appear normal or show diffuse brain abnormalities unspecific for a dementia subtype [9–11]. More advanced MRI techniques, such as diffusion tensor imaging (DTI) and resting state functional MRI (rs-fMRI), may aid differential diagnosis by detecting more subtle abnormalities that remain unrevealed using structural MRI [12].

DTI is used to assess white matter (WM) microstructure of the brain. Previous studies observed more pronounced microstructural WM abnormalities in bvFTD than in AD [13, 14] and suggested an anterior-posterior division of WM abnormalities in bvFTD and AD. Microstructural WM abnormalities are observed in the anterior brain regions in bvFTD, such as the cingulate cingulum (CGH), forceps minor (FMI) and uncinate fasciculus (UF), whereas microstructural WM changes in AD are localised in more posterior brain regions, such as the forceps major (FMA) and the hippocampal cingulum (CGH) [15–17]. Rs-fMRI is used to assess functional connectivity between grey matter (GM) regions that together form functional brain networks. A widely studied network is the default mode network (DMN), known to be affected in both AD and bvFTD [18]. Previous research has shown DMN differences between AD and bvFTD, specifically decreased DMN connectivity in AD and increased DMN connectivity in bvFTD—mostly in the posterior DMN.

Clinical diagnosis may especially benefit from objective quantitative measures derived from DTI and rs-fMRI in differentiating subtypes of dementia patients, and patients from healthy persons, using group-specific reference values. In this study, we explored group-wise quantitative measures of tract-specific WM microstructure and functional connectivity of the DMN in a small patient population, to provide an initial indication of their diagnostic utility for early-stage and over time differentiation of AD and bvFTD.

## Methods

### Participants

Patients with a suspected diagnosis of early AD or bvFTD were recruited soon after their initial visits to the Alzheimer Centre Rotterdam. Suspected AD or bvFTD diagnosis was established by a multidisciplinary team of neurologists, neuroradiologists, nuclear radiologists, geriatricians and neuropsychologists. Diagnostic criteria included patient complaints, medical history, neurological examination, radiological assessment and full cognitive assessment that were overall suggestive of AD or bvFTD and in line with the established diagnostic criteria for AD [2] and bvFTD [3]. Genetic testing was performed only in case of a positive family history for dementia. Six bvFTD patients included in this study had a genetic mutation (5 MAPT, 1 *C9ORF72*).

Inclusion criteria for this study were an age between 40 and 70 years; suspected diagnosis of early AD [2] or bvFTD [3]; a Mini-Mental State Examination [19] (MMSE) score of ≥ 20. Exclusion criteria were contraindications for MRI; an expected loss to follow-up within one year; other neurological disorders; a different cause of dementia; alternative psychiatric diagnosis; past or current substance abuse. Diagnosis of either AD or bvFTD was confirmed after at least one year follow-up. Patients underwent the Mini-Mental State Examination (MMSE) as part of their routine clinical diagnostic work-up. Healthy controls, matched for age and gender, and without neurological or psychiatric history, were recruited through advertisement. Controls underwent neuropsychological testing and the MMSE as part of this study to rule out cognitive impairment. The study was approved by the local medical ethics committee. All participants gave written informed consent.

### Image acquisition

MRI was performed on a 3 T Discovery MR750 system (GE Healthcare). See Table 1 for acquisition parameters. Patients underwent identical MRI protocols at baseline (T0) and at 1-year follow-up (T1). Controls underwent MRI at T0 only. For anatomical reference, a high-resolution three-dimensional (3D) inversion recovery (IR) fast spoiled gradient echo (FSPGR) T1-weighted (T1w) image was acquired. DTI scans were acquired with spin-echo echo planar imaging (EPI) and rs-fMRI scans with gradient echo EPI. For rs-fMRI, participants were instructed to think of nothing in particular, to focus on a fixation cross and to remain awake.

**Table 1:** Acquisition parameters

	T1w	DTI	fMRI
FOV (mm)	240	240	240
TE (ms)	3.06	84.5*	30
TR (ms)	7904	7930	3000
ASSET factor	2	2	2
Flip angle	12°	90°	90°
Acquisition matrix	240 × 240	128 × 128	96 × 96
Slice thickness (mm)	1	2.5	3
Volumes (slices per volume)	1 (176)	28 (59)	200 (44)
Duration (min)	4.41	3.50	10.00
Diffusion-weighted directions	n/a	25	n/a
Non-diffusion weighted images	n/a	3	n/a
Maximum *b* value (s/ mm^2^)	n/a	1000	n/a
TI (ms)	450	n/a	n/a

*T1w*, T1-weighted; *DTI*, diffusion tensor imaging; *fMRI*, functional magnetic resonance imaging; *FOV*, field of view; *TE*, echo time; *TR*, repetition time; *ASSET*, array spatial sensitivity encoding technique; *TI*, inversion time

*TE for DTI was set to minimum. This number represents the average TE. The range of TE was 81.9–90.8 ms

### Demographical analysis

Between-group differences in age were tested using ANOVA. Between-group differences in MMSE score were tested using the Welch-ANOVA and post hoc Games-Howell *t* tests, due to unequal variance across groups. Gender was compared across groups using the chi-square tests. Analyses were done using IBM SPSS Statistics 21.0 with a significance threshold of *p* < 0.05.

### GM volume analysis

GM volumes were calculated according to the methods described in Bron et al (2014) [20]. GM volumes were obtained from the T1w image using the unified tissue segmentation method of Statistical Parametric Mapping (SPM8), after which intracranial volume (ICV) was calculated. Then, GM volume was divided by ICV to correct for brain size. GM volume (%ICV) was compared for groups at T0 and at T1 using ANOVA and the post hoc Bonferroni tests.

### Microstructural WM analysis

WM tracts known to be associated with cognitive functions were selected for tractography: anterior thalamic radiation (ATR) [21], cingulum (CGH and CGC) [22], FMA [21], FMI [21, 23], inferior fronto-occipital fasciculus (IFOF) [24, 25], inferior longitudinal fasciculus (ILF) [24, 25], superior longitudinal fasciculus (SLF) [26, 27] and UF [22, 23, 25].

Tracts were generated using automated probabilistic tractography (AutoPtx) as implemented in FMRIB Software Library (FSL5) [28]. Median fractional anisotropy (FA), mean diffusivity (MD), radial diffusivity (RD) and axial diffusivity (AxD) were established for each tract. The quality of WM tracts was visually assessed. See supplement $1 for a full description.

The rate of change (T1-T0) was established for each diffusion measure for each tract. Then, diffusion measures at T0 and T1 and the rates of change were compared between groups using ANOVA and the post hoc Bonferroni *t* tests, unless an age effect was present. Age effects were investigated using linear regression and, if necessary, taken into account using ANCOVA. In case of unequal variances across groups, between-group differences were investigated using the Welch-ANOVA and post hoc Games-Howell *t* tests.

### Functional connectivity analysis

Using regions of interest (ROIs) of the Hammers atlas (30 atlases with 83 ROIs; http://brain-development.org/brain-atlases) [29], GM regions making up the DMN were selected for functional analysis: bilateral medial prefrontal cortex, lateral temporal cortex, inferior parietal lobule, precuneus and posterior cingulate cortex. ROIs were normalised to Montreal Neurological Institute (MNI) space.

Functional and structural data were pre-processed using SPM8 (supplement $2). This was followed by further pre-processing and analysis using the connectivity toolbox by Mantini [30, 31]. For each ROI, the average blood oxygenation level–dependent (BOLD) signal was calculated. Subsequently, the average BOLD signal of each ROI was correlated with all ROIs separately to assess functional connectivity. A Fisher’s *r*-to-*z* transformation was then applied to allow analysis of between-group functional connectivity differences. For both T0 and T1, functional connectivity between ROIs was established for each group using a random-effect analysis corrected for multiple comparisons (false discovery rate (FDR) < 0.001). Between-group differences at T0 and at T1 were assessed using ANCOVA (*p* < 0.05) with GM volume (%ICV) as covariate and as post hoc two-sample *t* tests (FDR < 0.05).

## Results

### Baseline (T0)

#### Participant characteristics

Baseline data from 11 AD patients, 12 bvFTD patients (9 for rs-fMRI) and 18 controls were used for the analysis (Table 2; see supplement $3 for exclusions).

**Table 2:** Demographic characteristics

Group	*N*	Mean age	Mean MMSE
BvFTD	12 (6 male)	60.3 (7.7)	26.6 (2.8)
BvFTD T1	6 (3 male)	64.0 (3.6)	–
AD	11 (8 male)	62.8 (5.0)	25.3 (2.0)
AD T1	11 (8 male)	63.3 (5.0)	–
Controls	18 (8 male)	59.8 (6.7)	29.1 (1.0)

*BvFTD*, behavioural variant frontotemporal dementia; *AD*, Alzheimer’s disease; *N*, sample size. Values given as mean (standard deviation). *MMSE*, Mini-Mental State Examination

Participants did not differ in age (*F*(2,38) = 0.498, *p* > 0.05), gender (*χ*^2^(2) = 2.288, *p* > 0.05) or education level (*χ*^2^(4) = 3.394, *p* > 0.05). Education level was unknown for 2 bvFTD patients, 1 AD patient and 1 control. MMSE score was different between groups (*F*(2,17.1) = 20.213, *p* < 0.001) and was lower in both patient groups compared with controls. MMSE score did not differ between AD and bvFTD.

#### GM volume

The total GM volume (%ICV) was significantly lower (*F*(2,38) = 13.837, *p* < 0.001) in bvFTD (0.30%ICV, standard deviation (SD) 0.04) compared with both AD (0.33%ICV, SD 0.03) and controls (0.36%ICV, SD 0.03), but not different between AD and controls.

#### WM microstructure

**Fig. 1:**
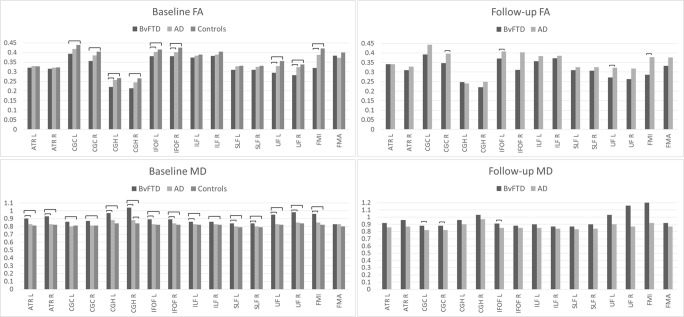
Mean fractional anisotropy (FA) and mean diffusivity (MD) at baseline and follow-up for individual white matter tracts shown per group (Alzheimer’s disease (AD), behavioural variant frontotemporal dementia (bvFTD) and controls). Significant differences (*p* < 0.05) between groups indicated by a horizontal line. CGH, cingulum (hippocampal); IFOF, inferior fronto-occipital fasciculus; ILF, inferior longitudinal fasciculus; SLF, superior longitudinal fasciculus; UF, uncinate fasciculus; FMI, forceps minor; FMA, forceps major; L, left; R, right

**Fig. 2:**
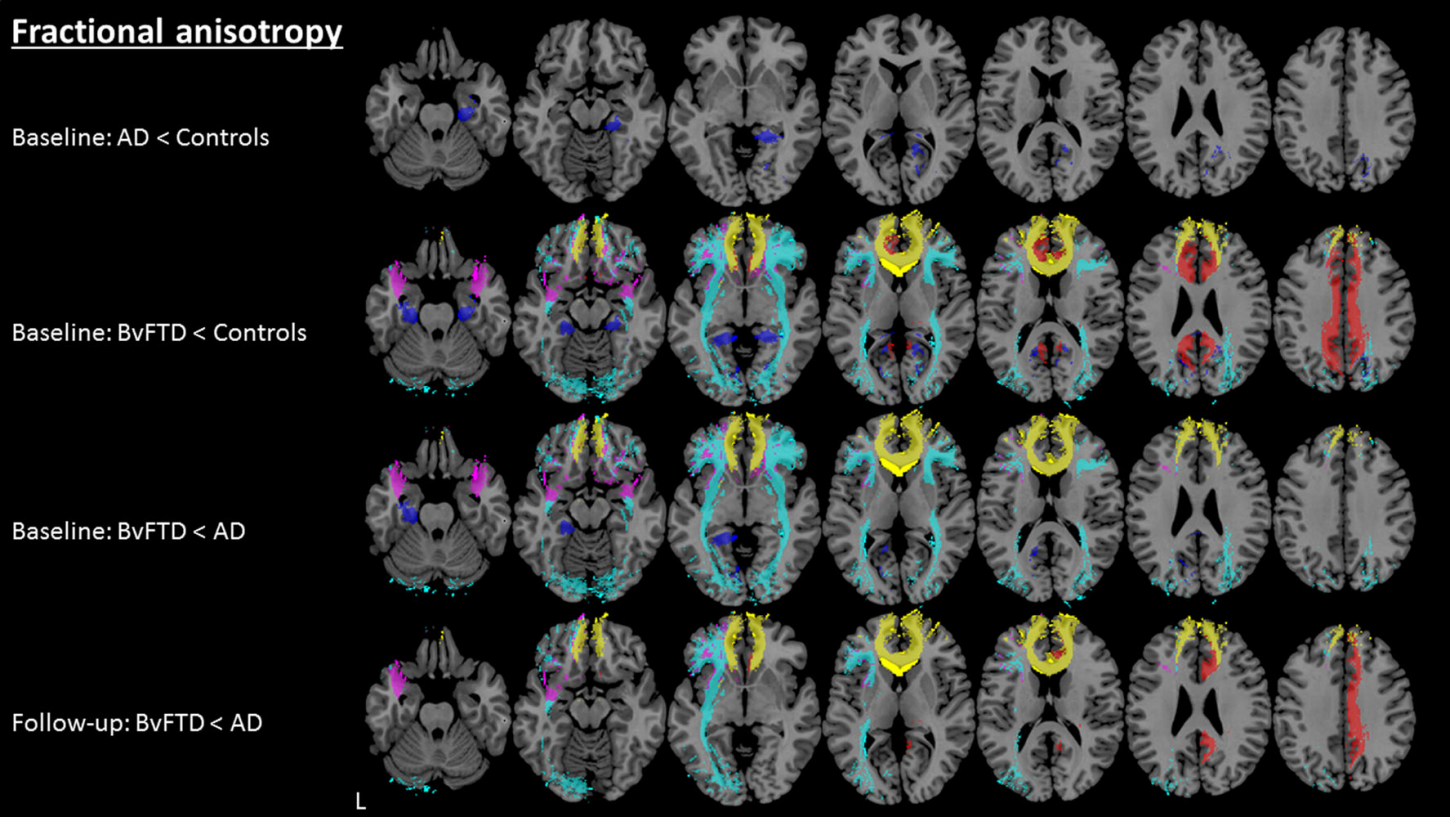
Between-group fractional anisotropy differences (*p* < 0.05) in white matter tracts at baseline and follow-up. Light blue, inferior-fronto occipital fasciculus; yellow, forceps minor; red, cingulate cingulum; dark blue, hippocampal cingulum; pink, uncinate fasciculus; AD, Alzheimer’s disease; bvFTD, behavioural variant frontotemporal dementia; L, left

WM tracts were correctly identified in all groups. AD in comparison with controls (Table 3) showed higher MD only in the *right* CGH. BvFTD in comparison with controls (Table 3; Fig. 1) showed lower FA and higher MD, RD and AxD in the *bilateral* CGH, IFOF, UF and FMI. Higher MD, RD and AxD in bvFTD compared with controls were additionally observed in the *bilateral* ATR, ILF and SLF. Further, lower FA and higher MD and RD in bvFTD compared with controls were observed in the *bilateral* CGC. BvFTD in comparison with AD (Table 3; Fig. 1) showed lower FA and higher MD, RD and AxD in the *bilateral* IFOF and UF and FMI. Higher MD, RD and AxD in bvFTD compared with AD were additionally observed in the *bilateral* ATR, SLF and *right* CGH. Lower FA in bvFTD compared with AD was additionally observed in the *left* CGH. Further, bvFTD in comparison with AD showed higher MD and RD in the *bilateral* CGH, higher MD and AxD in the left ILF and higher AxD in the *right* CGC. For an example of the affected WM tracts, see Fig. 2 where between-group differences in FA for individual WM tracts are shown.

**Table 3:** Mean FA, MD, RD and AxD for each WM tract, for bvFTD, AD and controls at T0. The numbers shown are multiplied by a factor 1000

WM tract	L/R	FA			MD			RD			AxD		
		BvFTD	AD	Controls	BvFTD	AD	Controls	BvFTD	AD	Controls	BvFTD	AD	Controls
ATR	L	320.44	328.95	327.82	***0.90***	0.83	0.81*	***0.73***	0.68	0.67	***1.27***	1.18	1.15*
ATR	R	315.64	320.27	322.81	***0. 93***	0.83	0.82	***0.76***	0.69	0.68	***1.31***	1.17	1.16*
CGC	L	**392.62**	417.66	438.54	***0. 86***	0.80	0.81	***0.67***	0.61	0.60	1.26	1.20	1.23
CGC	R	**355.58**	384.25	403.92	***0. 87***	0.81	0.81*	***0.70***	0.63	0.62	*1.23*	1.17	1.19
CGH	L	***221.51***	256.26	266.75	**0. 97**	0.88	0.84*	**0.85**	0.76	0.72*	**1.27**	1.19	1.13*
CGH	R	***213.55***	244.43	264.69	***1.04***	**0.88**	0.84*	***0.91***	0.76	0.72*	***1.34***	1.19	1.16*
IFOF	L	***380.55***	403.52	415.33	***0.89***	0.83	0.82	***0.68***	0.64	0.62	***1.30***	1.23	1.23
IFOF	R	***380.37***	402.05	425.34	***0.89***	0.84	0.82*	***0.70***	0.64	0.61	**1.31**	1.24	1.24*
ILF	L	373.10	383.60	388.35	***0.86***	0.83	0.82	**0.67**	0.64	0.63	***1.25***	1.21	1.19
ILF	R	382.13	389.16	403.75	**0.86**	0.83	0.82	**0.67**	0.64	0.62	**1.27**	1.22	1.20*
SLF	L	310.14	327.78	330.49	***0.84***	0.80	0.79	***0.69***	0.65	0.65	***1.16***	1.12	1.11
SLF	R	309.32	325.56	330.63	***0.84***	0.80	0.79	***0.70***	0.65	0.65	***1.17***	1.12	1.12
UF	L	***294.48***	329.90	355.41	***0.95***	0.83	0.82*	***0.79***	0.68	0.65*	***1.29***	1.17	1.17*
UF	R	***282.49***	323.75	338.02	***0.98***	0.85	0.84*	***0.83***	0.69	0.68*	***1.33***	1.19	1.19*
FMI	n/a	***319.98***	387.77	421.05	***0.96***	0.85	0.82*	***0.78***	0.66	0.62*	***1.30***	1.23	1.23
FMA	n/a	383.12	372.15	399.79	0.83	0.83	0.80	0.64	0.64	0.61	1.30	1.30	1.29

Italicised entries = vs other patient group *p* < 0.05; Bold entries = vs controls *p* < 0.05

*FA*, fractional anisotropy; *MD*, mean diffusivity; *RD*, radial diffusivity; *AxD*, axial diffusivity; *WM*, white matter; *bvFTD*, behavioural variant frontotemporal dementia; *AD*, Alzheimer’s disease; *ATR*, anterior thalamic radiation; *CGC*, cingulum (cingulate); *CGH*, cingulum (hippocampal); *IFOF*, inferior fronto-occipital fasciculus; *ILF*, inferior longitudinal fasciculus; *SLF*, superior longitudinal fasciculus; *UF*, uncinate fasciculus; *FMI*, forceps minor; *FMA*, forceps major; *L*, left; *R*, right

*Unequal variance, significance tested with the Welch-ANOVA

#### Functional connectivity

**Fig. 3:**
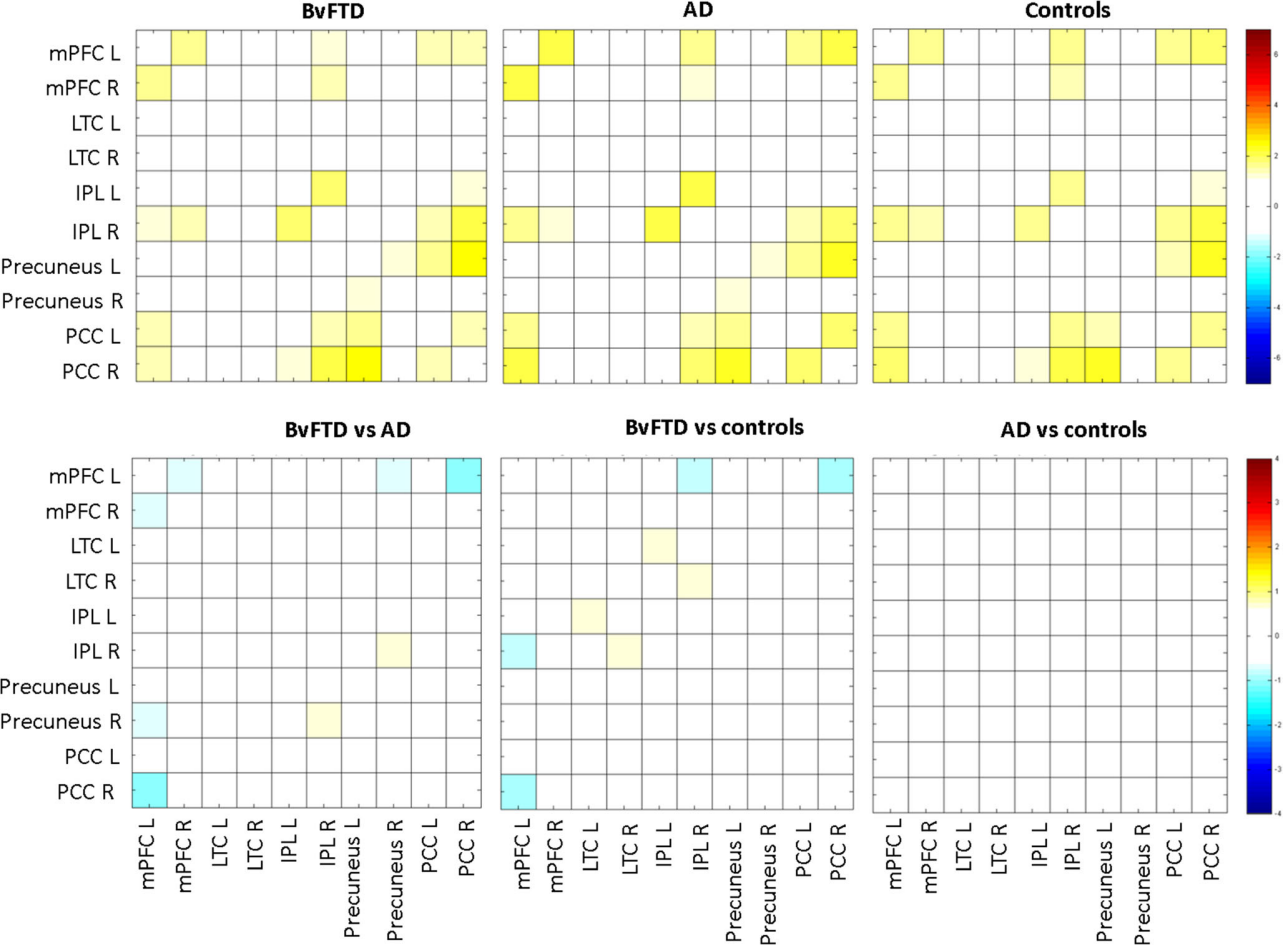
Non-significant default mode network (DMN) connectivity (*p* > 0.05; FWE_corrected_) at baseline (T0). DMN functional connectivity for behavioural variant frontotemporal dementia (bvFTD), Alzheimer’s disease (AD) and controls is shown in row one. Between-group differences for DMN functional connectivity are shown in row two. Colours represent the *t* values of between-region functional connectivity. mPFC, medial prefrontal cortex mPFC; LTC, lateral temporal cortex; IPL, inferior parietal lobule; PCC, posterior cingulate cortex; L, left; R, right

Significant DMN within-group functional connectivity and between-group functional connectivity changes were not observed (Fig. 3).

### Follow-up (T1)

#### Participant characteristics

Patients underwent a second MRI approximately 1 year later at T1 (mean 378 days). Six bvFTD patients did not undergo MRI at T1 and hence were excluded from T1 data analysis. Three of these patients had not been consented for a scan at T1 and three patients had progressed too severely. Data from 11 AD patients and 6 bvFTD patients were used for the analysis (Table 2). Participants did not differ in age (*t*(15) = 0.311, *p* > 0.05) or gender (*χ*^2^(1,15) = 0.88, *p* > 0.05).

#### GM volume

The total GM volume (%ICV) was different between AD and bvFTD (*t*(15) = − 2.266, *p* < 0.039) and was significantly lower in bvFTD (0.27%ICV, SD 0.05) than in AD (0.32%ICV, SD 0.04).

#### WM microstructure

WM tracts were correctly identified in both groups, except for in one bvFTD and one AD patient where eight tracts could not be reconstructed due to low data quality. Data for these tracts were not used.

BvFTD in comparison with AD (Table 4, Figs. 1 and 2) showed lower FA and higher MD and RD in the *right* CGC and *left IFOF*. Additionally, higher MD in bvFTD compared with AD was observed in the *left* CGC and lower FA in the *left* UF and FMI. No differences were observed in AxD.

**Table 4:** Mean FA, MD, RD and AxD for each WM tract, for bvFTD and AD at T1. The numbers shown are multiplied by a factor 1000

WM tract	L/R	FA		MD		RD		AxD	
		BvFTD	AD	BvFTD	AD	BvFTD	AD	BvFTD	AD
ATR	L	341.44	341.57	0.92	0.86	0.74	0.69	1.33	1.24
ATR	R	309.17	328.47	0.96	0.87	0. 79	0.71	1.37	1.23
CGC	L	392.49	443.31	*0.88*	0.82	Excluded	Excluded	1.28	1.22*
CGC	R	*346.15*	396.49	*0.88*	0.82	*0.71*	0.63	1.24	1.19
CGH	L	246.78	240.96	0.96	0.90	0. 82	0.78	1.27	1.20
CGH	R	220.07	248.85	1.03	0.97	0. 91	0.81*	1.31	1.33
IFOF	L	*370.40*	407.22	*0.91*	0.85	*0.71*	0.64	1.32	1.26*
IFOF	R	311.60	403.42*	0.88	0.85	0.67	0.65	1.30	1.26
ILF	L	357.26	383.46	0.90	0.85	0.71	0.65	1.28	1.23*
ILF	R	371.54	384.99	0.87	0.84	0.68	0.66	1.27	1.23
SLF	L	310.15	325.04	0.87	0.83	0.72	0.68	1.19	1.17
SLF	R	306.60	325.68	0.90	0.84	0.75	0.68	1.26	1.18
UF	L	*271.33*	323.24	1.03	0.90	0.87	0.73	1.35	1.25
UF	R	262.51	317.80*	1.16	0.87*	1.00	0.71*	1.53	1.21*
FMI	n/a	*285.17*	378.20	1.20	0.92	1.02	0.71	1.54	1.33
FMA	n/a	331.97	376.31	0.92	0.87	0.74	0.66	1.36	1.35

Italicised entries = vs other patient groups *p* < 0.05

*FA*, fractional anisotropy; *MD*, mean diffusivity; *RD*, radial diffusivity; *AxD*, axial diffusivity; *WM*, white matter; *bvFTD*, behavioural variant frontotemporal dementia; *AD*, Alzheimer’s disease; *ATR*, anterior thalamic radiation; *CGC*, cingulum (cingulate); *CGH*, cingulum (hippocampal); *IFOF*, inferior fronto-occipital fasciculus; *ILF*, inferior longitudinal fasciculus; *SLF*, superior longitudinal fasciculus; *UF*, uncinate fasciculus; *FMI*, forceps minor; *FMA*, forceps major; *L*, left; *R*, right

*Unequal variance. Significance tested with Welch-ANOVA

The rate of change between T1 and T0 (Table 5) of FA in the cingulum was different between bvFTD and AD. Specifically, the rate of change of FA in the *right* CGC was higher in bvFTD versus a lower change in AD, whereas the rate of change of FA in the *left* CGH was lower in bvFTD versus a higher change in AD. Additionally, the rate of change of AxD in the *right* IFOF was also different between bvFTD and AD; namely, it was lower in bvFTD versus higher in AD.

**Table 5:** Mean difference score (T1 minus T0) of FA, MD, RD and AxD for each WM tract, for bvFTD and AD. The numbers shown are multiplied by a factor 1000

WM tract	L/R	FA		MD		RD		AxD	
		BvFTD	AD	BvFTD	AD	BvFTD	AD	BvFTD	AD
ATR	L	9.80	8.75	0.03	0.03	0.02	0.02	0.05	0.05
ATR	R	− 9.84	5.81	0.02	0.04	0.03	0.02	0.03	0.06
CGC	L	− 5.59	20.23	0.01	0.01	0.01	0.0003	0.002	0.01
CGC	R	*− 29.77*	10.44	0.02	0.01	0.03	0.001	− 0.002	0.01*
CGH	L	*13.84*	− 19.95	0.03	0.01	0.02	0.01	0.04	− 0.003
CGH	R	− 5.78	− 1.84	0.03	0.08	0.04	0.05	0.02	0.14
IFOF	L	− 5.42	− 3.17	0.01	0.01	0.02	0.01	0.02	0.02
IFOF	R	− 73.01	− 4.87*	− 0.003	0.01	− 0.01	0.01	*− 0.008*	0.02
ILF	L	− 12.86	− 1.62	0.03	0.01*	0.03	0.01	0.04	0.02*
ILF	R	− 13.69	− 8.00	0.01	0.01	0.02	0.01	0.01	0.01
SLF	L	1.28	− 3.97	0.03	0.03	0.03	0.03	0.04	0.05
SLF	R	6.20	− 2.72	0.04	0.05	0.02	0.04	0.07	0.06
UF	L	− 25.28	− 12.54	0.09	0.07	0.10	0.06	0.08	0.07
UF	R	− 13.80	− 10.44	0.15	0.02*	0.15	0.02*	0.17	0.02*
FMI	n/a	− 30.22	− 13.49	0.23	0.07	0.23	0.06	0.24	0.10
FMA	n/a	− 37.71	− 3.78*	0.08	0.04	0.09	0.03*	0.04	0.06

Italicised entries = vs other patient group *p* < 0.05

*FA*, fractional anisotropy; *MD*, mean diffusivity; *RD*, radial diffusivity; *AxD*, axial diffusivity; *WM*, white matter; *bvFTD*, behavioural variant frontotemporal dementia; *AD*, Alzheimer’s disease; *ATR*, anterior thalamic radiation; *CGC*, cingulum (cingulate); *CGH*, cingulum (hippocampal); *IFOF*, inferior fronto-occipital fasciculus; *ILF*, inferior longitudinal fasciculus; *SLF*, superior longitudinal fasciculus; *UF*, uncinate fasciculus; *FMI*, forceps minor; *FMA*, forceps major; *L*, left; *R*, right

*Unequal variance, significance tested with Welch-ANOVA

#### Functional connectivity

**Fig. 4:**
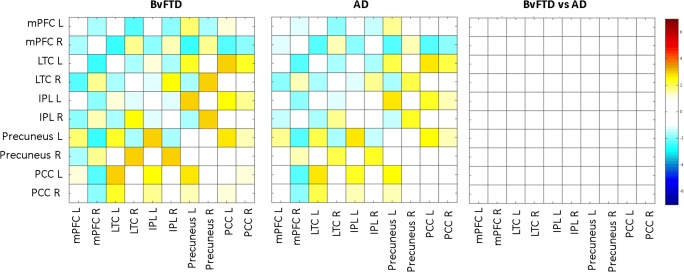
Non-significant default mode network (DMN) connectivity (*p* > 0.05; FWE_corrected_) at follow-up (T1). DMN functional connectivity for behavioural variant frontotemporal dementia (bvFTD) and Alzheimer’s disease (AD) is shown in columns one and two, respectively. Between-group comparison for DMN functional connectivity is shown in column three. Colours represent the *t* values of between-region functional connectivity. mPFC, medial prefrontal cortex mPFC; LTC, lateral temporal cortex; IPL, inferior parietal lobule; PCC, posterior cingulate cortex; L, left; R, right

Significant DMN within-group functional connectivity and between-group functional connectivity changes were not observed (Fig. 4). For this reason, added value and/or sensitivity of the rate of change analysis was not expected and therefore not performed.

## Discussion

In this study, we explored group-wise quantitative measures of tract-specific WM microstructure and functional connectivity of the DMN to provide an initial indication of their diagnostic utility for early-stage and over time differentiation of AD and bvFTD. Quantitative tract-specific microstructural WM abnormalities, but not quantitative functional DMN connectivity, may aid early-stage—and possibly over time—differential diagnosis of bvFTD and AD. Microstructural WM abnormalities were observed in widespread WM tracts in bvFTD, whereas they were only seen regionally in AD. Additionally, at follow-up, the differences in tract-specific microstructural WM abnormalities between bvFTD and AD became less pronounced, although they were still stronger in bvFTD. Despite these diminished differences, the rate of change was very similar between bvFTD and AD. It should be noted that this might be an underappreciation of differences due to bvFTD drop-out at follow-up.

### WM microstructure

#### Baseline

Quantitative microstructural WM abnormalities were seen in bvFTD and AD in different WM tracts, suggesting a differential diagnostic role for assessing diffusion values in a clinical context. Tract-specific WM microstructural abnormalities were evident in bvFTD in all WM tracts investigated, but most pronounced in the FMI, CGH, CGC, IFOF and UF. These tracts have been associated with cognitive domains characteristically affected in bvFTD [3]: the FMI with disinhibition and executive functioning [23, 32], CGH with memory and executive functioning [33, 34], CGC with cognitive control [35], IFOF with social cognition and emotional functioning [36, 37] and the UF with apathy, disinhibition and behavioural dyscontrol [23, 38, 39]. Unsurprisingly, WM abnormalities were not observed in the FMA, which is a posterior tract associated with visuospatial functioning [40], a domain generally preserved in bvFTD [41]. Microstructural WM abnormalities in bvFTD were evident in comparison with both AD and controls, but even more pronounced in comparison with the latter. This smaller difference between bvFTD and AD could indicate that changes in the WM in AD were already ongoing. AD in comparison with controls only showed microstructural abnormalities at baseline, specifically only in the CGH, suggesting the importance of this structure in AD. Previously, the CGH has been associated with memory functioning [42, 43], which is characteristically impaired in AD [2].

#### Follow-up

At follow-up, microstructural WM abnormalities were still more pronounced in bvFTD than in AD, but in fewer tracts, specifically in the *left* IFOF and UF and *right* CGC and FMI, suggesting these may be important for differentiating between bvFTD and AD at later stages. However, some caution is warranted, as six out of the twelve bvFTD patients did not return for follow-up. If these patients were more advanced than patients who participated at follow-up, not including them may have led to underappreciating WM abnormalities in bvFTD.

CGC involvement in bvFTD is in line with previous literature showing classification of bvFTD and controls to be best achieved using FA in the cingulum bundle [44]. The IFOF has been previously associated with a variety of cognitive domains, such as emotion recognition [37], executive functioning [34, 45] and processing speed [46], of which many have been associated with bvFTD [22, 47, 48]. The UF and FMI are both known to be important in bvFTD and are associated with characteristic bvFTD symptoms [23, 32, 38].

The rate of change, in terms of the difference in diffusivity abnormalities between baseline and follow-up, showed a faster decline in WM microstructure of the *right* CGC in bvFTD and the *left* CGH and *right* IFOF in AD. In line with the observed baseline and follow-up changes and previous literature [44], this may suggest a differential involvement of the cingulum, in which the anterior part is more affected in bvFTD and the posterior part in AD. This is supported by the macrostructural frontotemporal (anterior) and temporoparietal (posterior) involvement in, respectively, bvFTD and AD [40, 49]. The IFOF has been associated with many different cognitive functions—as described above—but has as yet not been specifically linked to AD or bvFTD. However, the observed *left* versus *right* IFOF involvement in, respectively, bvFTD and AD suggests that a disease-specific link may in fact be present.

#### Diffusion metrics sensitive to group differences

Differences between AD and bvFTD were most pronounced in MD and RD at baseline, and in FA (and to a lesser extent, MD and RD) at follow-up. First, this suggests that myelin abnormalities are more pronounced in bvFTD, as RD is thought to represent myelin damage [50] and AxD axonal loss [51]. As MD and FA are a combination of these measures, it may be that their changes observed here are induced by the changes in RD rather than AxD. Second, this suggests—also in line with previous literature [16, 44]—that FA, MD and RD are most sensitive to group changes and are therefore recommended for differentiation between AD and bvFTD.

### Functional connectivity

Functional DMN connectivity between AD and bvFTD was not different using our quantitative method. Previous literature observed differences in DMN regions using whole-brain independent component analysis [18, 52–54], such as increased parietal DMN connectivity in bvFTD and decreased parietal DMN connectivity in AD. However, in this study, we aimed to assess a different approach that may be used clinically, i.e. a quantitative measure of functional connectivity between DMN regions. The small sample size of this study, and thus low power, may have left possible group effects undetected. However, clinical use warrants sensitivity of measures at an individual patient level; hence, a low sensitivity of quantitative functional DMN connectivity does not seem suitable for individual diagnostics.

### Limitations

This study knows some limitations. First, the small sample size limits interpretation and generalizability of the results and it may particularly lead to underestimation of between-group differences. However, the findings of this study are in line with the literature and may indicate clinical utility of DTI, but not rs-fMRI, on an individual patient level. Second, sample size was smaller at follow-up than at baseline which may have induced an underappreciation of abnormality severity at follow-up and rate of change differences in the bvFTD patients.

### Conclusion

In this explorative group-wise study of quantitative brain MR measures in dementia, we aimed to provide an indication of their usefulness for differentiation between AD and bvFTD at multiple time points. We observed that quantitative tract-specific microstructural WM abnormalities, but not quantitative functional connectivity of the DMN, may aid differential diagnosis of bvFTD and AD at the early-stage and possibly over time. Specifically, pronounced microstructural WM changes in anterior WM tracts may differentiate bvFTD from AD, and microstructural WM abnormalities of the hippocampal cingulum, in the absence of other microstructural WM changes, may differentiate AD from bvFTD.

## Electronic supplementary material

ESM 1 (DOC 80.5 kb)

## Abbreviations

AD: Alzheimer’s disease
ATR: Anterior thalamic radiation
AxD: Axial diffusivity
BOLD: Blood-oxygenation-level dependent
bvFTD: Behavioural variant frontotemporal dementia
CGC: Cingulate cingulum
CGH: Hippocampal cingulum
DMN: Default mode network
DTI: Diffusion tensor imaging
EPI: Echo planar imaging
FA: Fractional anisotropy
FDR: False discovery rate
FMA: Forceps major
FMI: Forceps minor
FSL: FMRIB Software Library
FSPGR: Fast spoiled gradient echo
GM: Grey matter
ICV: Intracranial volume
IFOF: Inferior fronto-occipital fasciculus
ILF: Inferior longitudinal fasciculus
IR: Inversion recovery
MD: Mean diffusivity
MMSE: Mini-Mental State Examination
MNI: Montreal Neurological Institute
MRI: Magnetic resonance imaging
RD: Radial diffusivity
ROIs: Regions of interest
rs-fMRI: Resting state functional magnetic resonance imaging
SLF: Superior longitudinal fasciculus
SPM: Statistical Parametric Mapping
T1w: T1-weighted
UF: Uncinate fasciculus
WM: White matter
