# Supplementary material for: Exploring quantitative group-wise differentiation of Alzheimer’s disease and behavioural variant frontotemporal dementia using tract-specific microstructural white matter and functional connectivity measures at multiple time points
Source: Eur Radiol. 2019 Mar 11;29(10):5148–59. doi: 10.1007/s00330-019-06061-7 (PMC6719324; doi:10.1007/s00330-019-06061-7)
Supplement: Supplementary file 1 — (DOC 80.5 kb) [file 330_2019_6061_MOESM1_ESM.doc]

**Supplement**

$1. Microstructural WM analysis

Data was analysed using FMRIB Software Library (FSL5, Oxford, UK) [1–3]. Data were corrected for motion and eddy currents using Eddy Correct and then skull-stripped using BET [4].

Automated probabilistic tractography (AutoPtx) [5] was used to apply a tensor fit with DTIFIT [6], followed by a FNIRT registration and a BEDPOSTX probabilistic model fit for each participant. PROBTRACKX [6, 7] was then run for all selected WM tracts using default space seed, target, stop and exclusion masks available in AutoPtx [5], resulting in a participant-specific tract density image. Tract density images were normalised by dividing them by the number of fibres included in the tract-image and then binarised for WM tract segmentation, based on the best-fit segmentation thresholds established by De Groot et al. (2013) [5] (supplemental Table 1).

Quality of WM tracts was visually assessed. Diffusivity images were masked with thresholded tract images using FSLstats, to acquire median fractional anisotropy (FA), mean diffusivity (MD), radial diffusivity (RD) and axial diffusivity (AxD) for each tract.

Diffusion measures at T0 were compared between AD, bvFTD and controls using an ANOVA and post-hoc Bonferroni t-tests, unless an age effect was present. Age effects were investigated using linear regression and, if necessary, taken into account using an ANCOVA. TE variation - induced by the minimum setting during scanning - could not be accounted for by means of regression analysis as ANOVA revealed higher TE for AD than for bvFTD and controls. The effect of varying TE is expected to be small; however a possible bias may have been introduced [8]. In case of unequal variances across groups, between-group differences were investigated using a Welch-ANOVA and post-hoc Games-Howell t-tests. Second, diffusion measures at T1 were compared between AD and bvFTD, following the baseline analysis approach. Two diffusion measures for separate WM tracts with both unequal variance across groups and age effect were excluded from the analysis. Third, AD and bvFTD diffusion measures at T0 were subtracted from T1 measures to establish the difference score per diffusion metric per tract. These difference scores (rate of change) were then compared between AD and bvFTD using the baseline analysis approach.

Supplemental Table 1. Tractography tract thresholds based on De Groot et al. (2013), but multiplied with a factor of eight due to the resolution difference.

| **White matter tract** | **Tractography threshold** |
| --- | --- |
| Anterior thalamic radiation (ATR) | 0.016 |
| Cingulum (cingulate gyrus) | 0.08 |
| Cingulum (hippocampal region) | 0.16 |
| Forceps major (FMa) | 0.04 |
| Forceps minor (FMi) | 0.08 |
| Inferior fronto-occipital fasciculus (IFOF) | 0.08 |
| Inferior longitudinal fasciculus (ILF) | 0.04 |
| Superior longitudinal fasciculus (SLF) | 0.008 |
| Uncinate fasciculus (UF) | 0.08 |

$2. Functional connectivity analysis

Functional and structural data were first pre-processed using Statistical Parametrical Mapping (SPM8, Wellcome department, London, UK. Spatial pre-processing consisted of manual realignment of functional and structural data to the anterior commissure, realignment of functional data, co-registration of functional and structural data, segmentation of structural data into GM and WM with a light clean, and normalisation to MNI space with a resampling size of 3mm^3^ for functional and 1mm^3^ for structural data. Further pre-processing and analysis were performed using the connectivity toolbox by Mantini [9, 10]. Functional data were scrubbed, smoothed with a Gaussian kernel of 5mm^3^ and corrected for motion, WM and cerebrospinal fluid signals. Additionally, band-pass filtering (0.009-0.08 Hz) was applied.

$3. Participant characteristics at T0: exclusions

Eleven AD patients, 12 bvFTD patients and 21 controls were included in the study. Three

controls were excluded due to incidental structural imaging findings. Three bvFTD patients were

excluded from the functional connectivity analysis due to missing rs-fMRI data.

**References**

1. Jenkinson M, Beckmann CF, Behrens TEJ, et al (2012) FSL. Neuroimage 62:782–790
2. Woolrich MW, Jbabdi S, Patenaude B, et al (2009) Bayesian analysis of neuroimaging data in FSL. Neuroimage 45:S173-S186
3. Smith SM, Jenkinson M, Woolrich MW, et al (2004) Advances in functional and structural MR image analysis and implementation as FSL. Neuroimage 23:S1:S208-S219
4. Smith SM (2002) Fast robust automated brain extraction. Hum Brain Mapp 17:143–155
5. de Groot M, Vernooij MW, Klein S, et al (2013) Improving alignment in Tract-based spatial statistics: evaluation and optimization of image registration. Neuroimage 76:400–411
6. Behrens TEJ, Woolrich MW, Jenkinson M, et al (2003) Characterization and propagation of uncertainty in diffusion-weighted MR imaging. Magn Reson Med 50:1077–1088
7. Behrens TEJ, Berg HJ, Jbabdi S, et al (2007) Probabilistic diffusion tractography with multiple fibre orientations: What can we gain? Neuroimage 34:144–155
8. Kim SJ, Choi CG, Kim JK, et al Effects of MR parameter changes on the quantification of diffusion anisotropy and apparent diffusion coefficient in diffusion tensor imaging: evaluation using a diffusional anisotropic phantom. Korean J Radiol 16:297–303
9. Ebisch SJH, Gallese V, Willems RM, et al (2011) Altered intrinsic functional connectivity of

anterior and posterior insula regions in high-functioning participants with autism spectrum

disorder. Hum Brain Mapp 32:1013–28 . doi: 10.1002/hbm.21085

1. Verly M, Verhoeven J, Zink I, et al (2014) Altered functional connectivity of the language network in ASD: Role of classical language areas and cerebellum. NeuroImage Clin 4:374–382. doi: 10.1016/j.nicl.2014.01.008
